# Age-Dependent Behavioral and Metabolic Assessment of *App*^*NL*−*G*−*F*/*NL*−*G*−*F*^ Knock-in (KI) Mice

**DOI:** 10.3389/fnmol.2022.909989

**Published:** 2022-07-29

**Authors:** Shanshan Wang, Taiga Ichinomiya, Paul Savchenko, Swetha Devulapalli, Dongsheng Wang, Gianna Beltz, Takashi Saito, Takaomi C. Saido, Steve L. Wagner, Hemal H. Patel, Brian P. Head

**Affiliations:** ^1^Veterans Affairs San Diego Healthcare System, San Diego, CA, United States; ^2^Department of Anesthesia, University of California, San Diego, San Diego, CA, United States; ^3^Department of Anesthesiology and Intensive Care Medicine, Graduate School of Biomedical Sciences, Nagasaki University, Nagasaki, Japan; ^4^Laboratory for Proteolytic Neuroscience, RIKEN Brain Science Institute, Saitama, Japan; ^5^Neurosciences Department, University of California, San Diego, San Diego, CA, United States

**Keywords:** APP, knock-in, mitochondria dysfunction, cognition deficit, fusion/fission dynamic

## Abstract

Mitochondria play a crucial role in Alzheimer's disease (AD) onset and progression. Traditional transgenic AD mouse models which were widely used in the past decades share a common limitation: The overexpression of APP and overproduction of amyloid-beta (Aβ) are accompanied by other APP peptide fragments, which could introduce artificial and non-clinically relevant phenotypes. Here, we performed an in-depth and time-resolved behavioral and metabolic characterization of a clinically relevant AD mouse model engineered to express normal physiological levels of APP harboring humanized Swedish (K670N/M671L), Beyreuther/Iberian (I716F), and Arctic (E693G) mutations (*App*^*NL*−*G*−*F*/*NL*−*G*−*F*^), termed APP knock-in (APPKI) mice. Our result showed that APPKI mice exhibited fear learning deficits at 6-m age and contextual memory deficit at 12-m age. Histopathological analysis revealed mild amyloidosis (6E10) accompanied by microgliosis (Iba1) as early as 3 months, which progressed significantly together with significant astrocytosis at 6 and 12 m. We further analyzed hippocampal mitochondrial dysfunction by multiple assays, while 3-m APPKI mice brain mitochondrial function remains a similar level as WT mice. Significant mitochondrial dysfunction characterized by decreased ATP production and higher membrane potential with subsequent overproduction of reactive oxygen species (ROS) was observed in mitochondria isolated from 7-m APPKI mice hippocampal tissue. Morphologically, these mitochondria were larger in volume with a decreased level of mitochondrial fusion protein mitofusin-2 (MFN2). At 12 months, APPKI mice exhibit a significantly decreased total mitochondrial oxygen consumption rate (OCR) in isolated hippocampal mitochondria detected by high-resolution respirometry. These data indicate early mitochondrial dysfunction in the brain at pre-symptomatic age in the *App*^*NL*−*G*−*F*/*NL*−*G*−^mice, which may play a key role in the progression of the disease. Moreover, the identified behavioral and bioenergetic alterations in this clinically relevant AD mouse model provide a valuable tool to optimize the temporal component for therapeutic interventions to treat AD.

## Introduction

In the last several decades, traditional transgenic mouse models of Alzheimer's disease (AD), such as APP/PSEN1 and 5xFAD, overexpress human mutant amyloid precursor protein (APP) to increase pathological amyloid-β peptide and were widely used in research to develop therapeutic approaches for AD. Although those traditional models exhibit critical features of amyloid pathology, the transgenic overexpression of human mutant APP also results in excessive production of other non-clinically relevant APP peptide species (Saito et al., 2014). While the exact biological role of APP is yet to be identified, multiple cellular functions have been suggested. Therefore, the non-physiological APP overexpression may cause artificial phenotypes and hamper data interpretation. The novel *App*^*NL*−*G*−*F*/*NL*−*G*−*F*^ (APPKI) mouse recently developed by Saito Lab only expresses endogenous levels of APP (Saito et al., 2014) that harbor humanized Swedish (K670N/M671L), Beyreuther/Iberian (I716F), and Arctic (E693G) mutations. This model exhibits APP expression and processing identical to that observed in human brains of AD subjects and develops progressive Aβ deposition. Thus, it has been more often used for identifying mechanisms of Aβ amyloidosis and potential treatment.

Mounting evidence suggests that mitochondrial dysfunction plays a fundamental role in the pathogenesis of AD (Wang et al., 2020). In highly metabolic organs like the brain, mitochondria play a central role in adapting to various stresses by providing the fuel for homeostasis. In the neuronal system, mitochondria are not only involved in presynaptic transmitter synthesis and release (Lee and Peng, 2008; Subramanian and Jonas, 2021), but they also dynamically interact with other cellular organelles to coordinate a stress response under pathological conditions (Tucker et al., 2016; Restelli et al., 2018). Mitochondrial dysfunction has been found as one of the intracellular processes severely compromised at the early stage of AD. Many mitochondrial parameters including bioenergetics, fusion/fission dynamic, mitophagy, and ROS generation were found to occur prior to clinical symptomatic stage and may actively contribute to the disease onset and progression of AD (Iturria-Medina et al., 2017). Thus, it is not surprising that mitochondria have been considered as a therapeutic target in AD (Gibson et al., 2008; Mao et al., 2012).

While several studies have demonstrated the characteristic of APP-related pathology of the newly developed APPKI mice, none have investigated the metabolic dysfunction of this strain. Considering that impaired energy metabolism precedes the clinical onset of AD, mitochondrial dysfunction has been considered as an early event in the development of AD (Swerdlow, 2018). The present study was designed to perform a comprehensive characterization of metabolic profile for the *APPKI* mice combined with the age-dependent evaluation of cognitive function, APP pathology, and neuroinflammation.

By utilizing multiple behavioral paradigms, the present study has shown that *APPKI* mice exhibit significant learning deficits at 6 months (m) and severe learning and memory deficits at 12 m. Furthermore, brains from APPKI mice also exhibit a wide range of mitochondrial dysfunction including alterations in mitochondrial membrane potential, ATP production, and ROS production. Furthermore, immunoblot analysis of dynamic proteins revealed the decreased expression of mitochondrial fusion protein (MFN2) at the early stage of the disease, while other mitochondrial dynamic proteins still maintained normal levels. This latter finding may contribute to the impaired mitochondrial bioenergetics and cognitive deficits observed at the same age. In summary, our results reveal age-dependent behavioral and bioenergetic alterations in this clinically relevant AD transgenic mouse model.

## Materials and Methods

### Animals

APPKI mice were derived from the Riken Institute colony (Japan) (Saito et al., 2014). C57BL/6 mice were purchased from Jackson Laboratory (Bar Harbor, ME, USA) and bred in-house. All animal protocols were approved by the Veterans Administration San Diego Healthcare System Institutional Animal Care and Use Committee (#20-030). Mice were reared (three–five/cage) with free access to food and water.

### Behavioral Analysis

Spatial learning and memory were assessed using Morris water maze (MWM) and fear conditioning (FC) (Wang et al., 2021b). Locomotor activity was assessed using open-field (OF) tests (Wang et al., 2021b). Fear conditioning test was performed at the end of all behavioral tests. All behavioral tests were performed for 3-, 6-, and 12-m-old male mice. Due to the stressful nature of fear conditioning test and eliminating artifacts by repeating these learning tasks, we used different cohorts including APPKI mice and age-matched WT for each time point. Each round of behavioral battery took 3 weeks in total. All behavioral tests were conducted by the researcher that was blinded to the experimental groups.

### Open Field

The open-field (OF) test was used to assess exploratory and general activity (Wang et al., 2021b). The apparatus consists of a square-shaped open arena (41 × 41 × 34 cm enclosures) with white acrylic walls. Mice were habituated to the testing room and subjected to a 10-min test session. The mice were placed in the open-field center with a bright light that illuminated the whole arena, to elicit a feeling of openness in the center of the maze (Seibenhener and Wooten, 2015). The time spent in the center area, the moving velocity, and the distance are used to evaluate the exploratory activity and general motor activity as previously described (Wang et al., 2021b). Locomotion was recorded and analyzed by a computerized video tracking system (Noldus XT 7.1, Leesburg, VA).

### Morris Water Maze

Morris water maze (MWM) test was performed as previously described (Arner et al., 2018). The arena consisted of a 180-cm diameter pool filled with opaque water (24°C). A different shaped marker was placed on walls for a visual clue. The platform was submerged in the water throughout the whole training process. The training session consisted of a 7-day training regime. For the first 3 days of the training session, the mice were initially trained to reach the submerged platform with a small visible flag attached on the platform above the water surface. For the following 4 days, the flag was removed and the mice were trained to reach the submerged hidden platform. These trainings were performed in three daily trials with 2-min intervals. The mice that could not find the platform in 90 s were placed on it for 30 s. Two alternative starting points with the same distance from the platform were used rotationally throughout the training. On day 8, the platform was removed, and the time spent in the correct quadrant during the test (40 s) was recorded. A visual performance test with the platform was performed on the same day following the non-platform trial. Mice that failed to reach the platform were scored and excluded from the final analysis. The latency to escape and distance were recorded with a Noldus Instruments EthoVision video tracking system (San Diego Instruments, San Diego, CA, USA).

### Fear Conditioning

Fear conditioning (FC) was used to assess learning and hippocampus-dependent contextual memory and hippocampus-independent cue memory and performed as previously described (Wang et al., 2021b). In brief, the presentation of unconditioned stimuli (US) (electric foot shock) and conditioned stimuli (CS) (auditory tone) was controlled using Med Associates Inc. (St. Albans, Vermont), and the movement was monitored by video. A total of five electric foot shocks were used on day 1 of the test. On day 2, mice were placed in the same test box to measure hippocampus-dependent contextual memory. On day 3, mice were placed in a different box with distinguished odor and five auditory tones were played to measure cue memory. The freezing percentage (a ratio of freezing duration during each session) was determined using Video Freeze (Med Associates Inc.; San Diego Instruments, San Diego, California) and analyzed as previously described (Wang et al., 2021b). Mice that failed to show increased freezing during day 1 were considered to fail to learn and were excluded from the analysis of memory recall on both day 2 and 3.

### High-Resolution Mitochondrial Respirometry

To avoid potential effects induced by behavioral tasks, we waited 2–4 weeks after the last behavioral test before organ harvesting for mid-stage (6-m) and late-stage (12-m) cohorts. A separate cohort of 3-m APPKI mice were used in this experiment. Mice were euthanized, and hippocampi were collected and processed immediately on ice for mitochondrial function assessment (Kilbaugh et al., 2015; Wang et al., 2021a). An Oroboros O2k respirometer was used to assess oxygen consumption rate. In brief, oxygen polarography was performed at 37°C, and oxygen flux per tissue mass was recorded in real time using DatLab software (Oroboros Instruments, Innsbruck, Austria). Hippocampal tissue was homogenized in cold Miro5 media on ice containing: 0.5 mM EGTA, 3 mM MgCl2, 60 mM K-lactobionate, 20 mM taurine, 10 mM KH2PO4, 20 mM HEPES, 110 mM sucrose, and 1.0 g/L fatty acid-free BSA, pH 7.1. One milligram of hippocampi lysate was loaded into each of the 2-mL respirometer chamber containing MiRO5 and kept at 37 °C during the measurement. After 10 min of equilibration, OXPHOS—capacity of complex I (CI OX)-linked activity was measured by adding CI-linked substrate pyruvate (5 mM), glutamate (10 mM), malate (0.5 mM), and ADP (2.5 mM). Mitochondrial quality and outer membrane integrity were verified by adding exogenous cytochrome C (10 uM). Leak state was calculated by mitochondria. The maximum oxidative phosphorylation capacity (CI and II OXPHOS) of CI- and II-linked activity was measured by the addition of succinate (10 mM), and the maximum uncoupled capacity (mUC) was determined following stepwise titration of 2-[2-[4-(trifluoromethoxy)phenyl]hydrazinylidene]-propanedinitrile (FCCP). Rotenone (0.5 uM) was added to inhibit complex I and the remaining measured complex II-driven respiration (CII ETS). Finally, complex III inhibitor antimycin-A (1 ug/ mL) was added to measure the residual oxygen consumption (Rox) that is independent of ETS. Oxygen concentration in the chamber was kept at more than 150 uM until the end to avoid the limitation of respiration. Each biological replicate (*n* ≥ 6) had a minimum of two technical replicates for the following experiment, and the average value was used for the final analysis. Oxygen consumption rate was obtained using DatLab 7 software.

### Bioenergetic Analysis of Isolated Hippocampal Mitochondria

Freshly extracted hippocampi (from both hemispheres) were rinsed with PBS to remove the blood and homogenized with mitochondrial isolation buffer. The lysate was then processed as described in the manufacturer's instructions (Mitochondrial Isolation Kit for Tissue, Thermo Fisher, #89801). In brief, the hippocampus was homogenized in 800 uL isolation buffer A containing fatty acid-free BSA (4 mg/mL). Eight hundred microliters of isolation buffer C was added to the lysate and mixed well by inverting tubes several times. Large debris and nuclei were removed by centrifugation for 10 min at 700 g at 4°C. The supernatant then underwent another round of centrifugation at 3,000 g for 15 min at 4°C to get a mitochondrial pellet. Five hundred microliters of wash buffer was added gently on the top of the pellet for surface washing by centrifugation at 12,000 g for 5 min. The mitochondrial pellet was then resuspended in 200 ul of mitochondrial isolation buffer for the following assay.

### Measurement of ATP Production

ATP production was quantitatively measured using a luciferin-/luciferase-based assay. The ATP determination kit containing substrate, enzyme, and buffer was purchased from Thermo Fisher Scientific (cat #A22066, Invitrogen) (Calkins et al., 2011). In brief, a TECAN Infinite M200 spectrofluorometer was used to measure light output at 560 nm, and ATP levels were quantitated according to the standard curve that was generated by a standard ATP solution. Two microliters of suspended mitochondrial sample was loaded into the 96-well dark plate followed together with 90 ul of standard reaction solution. Two microliters of ADP (10 mM) was then loaded, and ATP productions were quantified at 0, 20, 40, and 60 min after the addition of ADP. The plate was maintained at 37 °C to allow the reaction to proceed during the whole measurement. The final ATP production rate was normalized by protein concentration determined by the Bradford assay. Each biological replicate (*n* ≥ 3) had three technical replicates for the following experiment.

### Measurement of Oxidative Stress and Mitochondrial Membrane Potential

Thirty microliters of suspended mitochondria was used for each staining. MitoTracker Red FM (MTR, 100 nM, Invitrogen) was used to confirm mitochondrial identity. Then, TMRE (1 uM) was used to assess mitochondrial membrane potential, reflecting the extent to which oxygen consumption results in the generation of an effective proton gradient. Mito-Sox (20 uM) was used to quantify mitochondrial superoxide, and DCFDA (20 uM) was used to quantify mitochondrial ROS. TMRE, Mito-Sox, and DCFDA were added separately with MTR into 25 ug of suspended mitochondria for each staining mix to measure membrane potential (ΔΨm), superoxide, and reactive oxygen species, respectively. The dyes selected in this experiment exhibited distinct spectral properties with minimal to no overlap. After incubation at 37°C for 45 min, the samples were washed two times to remove excess dye and proceeded to flow cytometry. The forward scatter (FSC: 450) and side scatter (SSC: 250) voltages were adjusted for small events. FSC-A (area), FSC-W (width), and FSC-H (height) were collected to exclude doublets and gate mitochondrial singlets as described in result. MTR staining was then used to gate intact mitochondrial population as described (Pickles et al., 2013, 2014). Mito-Sox, DCFDA, and TMRE staining was then assessed in the gated MTR-positive singlets. A minimum of 10,000 events were acquired for each sample on cytoflow (Beckman Coulter). TMRE, Mito-Sox, and DCFDA intensity was assessed in each staining mix, and data were analyzed with FlowJo (Treestar, Ashland, Oregon, USA).

### Immunoblotting

To acquire specimens for immunoblot, the mice were perfused with cold PBS and one side of the hippocampus was dissected immediately (Wang et al., 2021b). Hippocampal tissue was homogenized at 4°C in RIPA buffer (containing protease and phosphatase inhibitors) and followed by sonication (10 s × three times with a 10-s interval). Ten micrograms of protein was loaded for immunoblot as previously described (Wang et al., 2021b). GAPDH was used as a loading control for total lysate immunoblots. Primary antibodies used were as follows: p-DRP(Ser616) (Thermo Fisher; #PA5-64821, 1:500), DRP1 (Cell Signaling, #8570, AB_10950498, 1:1000), mitofusin-1 (Abcam, #104274, 1:500), mitofusin-2 (Cell Signaling, #9482, AB_2716838, 1:1000), p-MFF (Ser146) (Cell Signaling, #49281, 1:1000), MFF (Cell Signaling, #84580, AB_2728769, 1:1000), Tom20 (Cell Signaling, #42406, AB_2687663, 1:1000), VDAC1 (Santa Cruz, #sc-390996, 1:50), OXPHOS (Abcam, #ab110413, AB_2629281, 1:2000), and GAPDH (Cell Signaling #2118s; AB_561053, 1:1,000) overnight at 4°C followed by incubation with an IR-dye-labeled secondary antibody for 1 hour and measured with LiCor Odyssey followed by densitometric analysis.

### Immunofluorescence Microscopy

The remaining hippocampal hemisphere (the same mice used for immunoblot) after saline perfusion was dissected immediately and subjected to postfix in 4% of PFA for a week (Wang et al., 2021b). The brain was then dehydrated in 30% sucrose at 4°C until it sank to the bottom. Brain sections with a thickness of 40 um were generated by cryosection and incubated in 98% formic acid for 5 min to expose the epitope before the blocking process. Slices were incubated with Alexa Fluor 488 anti-β-Amyloid 1-16 antibody (6E10, 1:200, BioLegend, #39347, USA), GFAP (1:500, Abcam, #7260, USA), and Iba1 (1:500, WAKO, #019-19741, Japan) at 4°C overnight following species-specific fluorescent secondary antibody in the dark for 1 h at room temperature. Slides were then preserved with an anti-fade DAPI-mounting medium and proceeded to visualize using an All-in-one fluorescence microscope (#BZ-X700, Keyence, IL, USA). The amyloid burden of the hippocampal area was quantified as 6E10 area normalized by the whole hippocampal area. GFAP staining and Iba1 staining were quantified by the mean fluorescence intensity (MFI) and was normalized to 7-m WT control to assess gliosis and astrocytosis. Each biological replicate contains three to five slices.

### Statistical Analysis

All data were analyzed using GraphPad Prism 8 (GraphPad Software, La Jolla, California, USA). For comparison between two groups, data were checked for normal distribution and analyzed by either Student's *t*-test or two-way ANOVA followed by Bonferroni's *post-hoc* analysis or LSD multiple *post-hoc* analysis as described. All data are presented as mean ± standard error of the mean (SEM), *n* corresponds to the number of each independent experiment, and significance was assumed when *p* < 0.05. Experimental groups were blinded to the observer, and the code was broken for analysis.

## Results

### Age-Dependent Cognition Decline in the APPKI Mice

**Figure 1:**
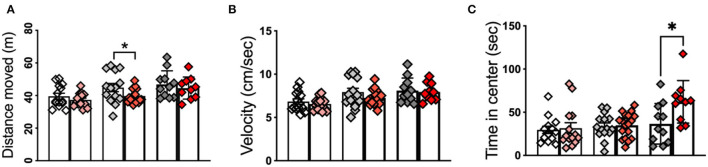
Open-field test of 3-m, 6-m, and 12-m APPKI mice with age-matched WT mice. **(A)** Open-field test revealed a reduction in locomotor/exploratory activity in 6-m APPKI mice compared with age-matched WT. **(B)** No significant difference was observed in moving velocity in the APPKI mice at all three age points. **(C)** Open-field test revealed 12-m APPKI mice spent a longer time in the center area of the open-field arena. Data are presented as mean ± SEM; *n* = 10–20 mice per group. Data were analyzed using Student's *t*-test between WT and APPKI group from the same time point. Significance was assumed when *p* < 0.05. *p < 0.05.

OF test revealed significant differences in the duration that APPKI mice spent in the center. At 6 m of age, APPKI mice moved significantly less distance compared with WT, suggesting a decreased exploration activity in the 6-m APPKI mice (Figure 1A). This is consistent with findings from Sergeant Lab that reported a reduction in open-field activity in the absence of memory deficit in the APPKI mice (Whyte et al., 2018). No significant difference was observed in the moving velocity at all three time points, although we see a decreasing trend between 6-m APPKI mice and age-matched WT (*p* = 0. 1541, two-tailed Student's *t*-test) (Figure 1B). Further analysis of duration in the center area of the open arena revealed no significance observed at 3 and 7 m, while 12-m APPKI mice exhibited a longer exploration time in the center compared with age-matched WT mice (Figure 1C). Rodents typically prefer not to be in the center (open area of the arena) and tend to move closer to the walls (thigmotaxis), since the center poses the risk of being exposed to a potential predator. Increased time spent exploring the center area could be interpreted as an indicator of risk-taking behavior or a lack of inhibition (Momeni et al., 2014). These data show that 12-m APPKI mice exhibit a significant disinhibition in the open field by spending more time in the center. No significant difference was observed in total distance traveled or velocity in APPKI mice at all three age points (Figures 1B,C).

**Figure 2:**
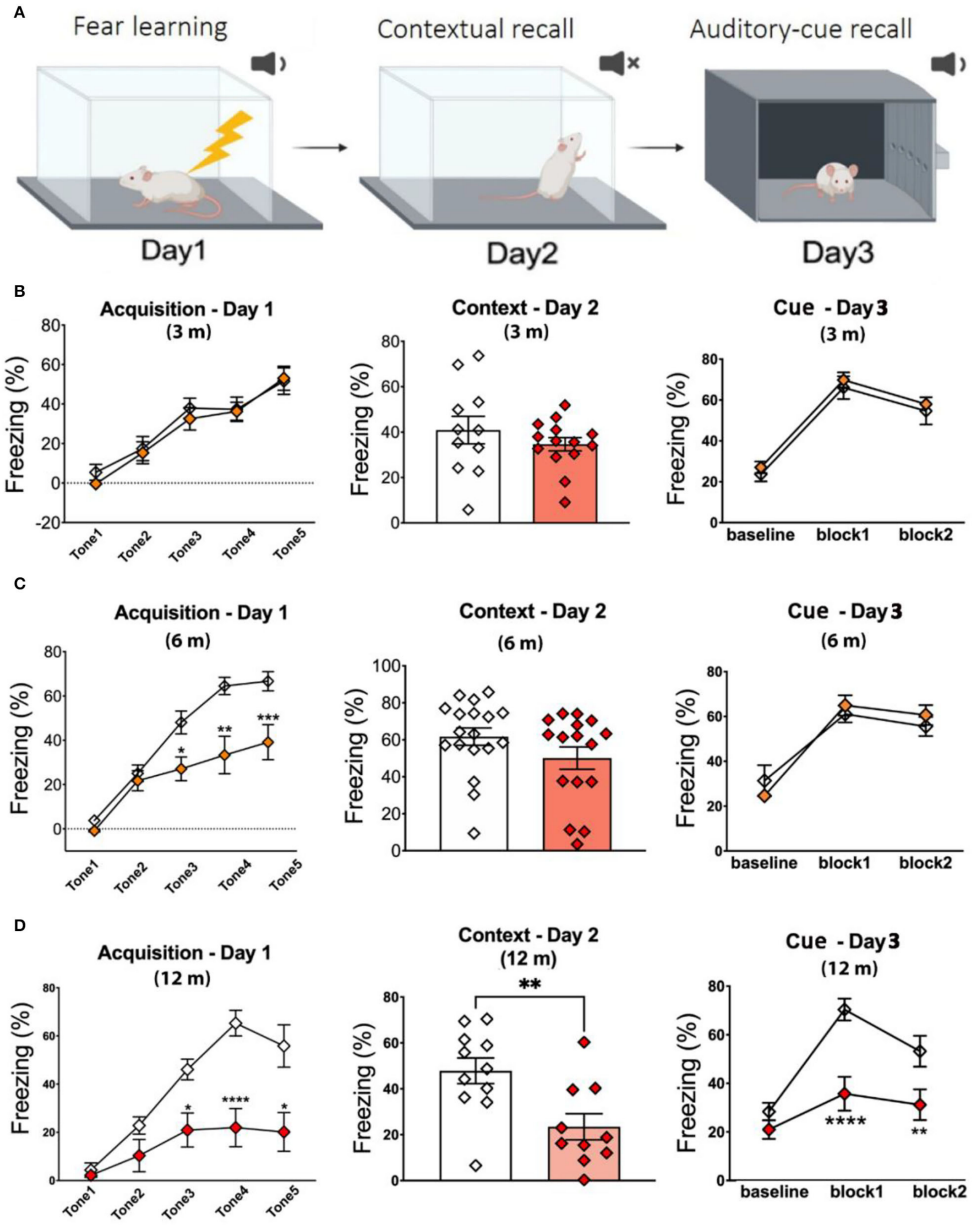
Fear learning and memory deficit in APPKI mice. **(A)** Graphic illustration of fear conditioning test. **(B)** No deficit was observed in fear conditioning of 3-m APPKI. **(C)** Deficit in fear leaning was observed in 7-m APPKI. **(D)** Deficit from learning to contextual memory and cue memory was observed in 12-m APPKI. Data are presented as mean ± SEM; *n* = 10–16 mice per group. Data were analyzed using Student's *t*-test for day-2 analysis and one-way ANOVA followed by Bonferroni's multiple comparisons test for day-1 and day-3 analysis. Significance was assumed when *p* < 0.05. *p < 0.05, **p < 0.01, ****p < 0.001.

Fear conditioning (FC) test has evolved as a standard procedure for testing contextual fear learning and memory (Wotjak, 2019). As shown in Figure 2A, day 1 is used to assess fear learning, while day 2 (context) and day 3 (cue) assess hippocampus-dependent contextual memory recall and non-hippocampus-dependent cue memory recall, respectively. No significant changes in percent freezing were observed on days 1–3 in 3-m APPKI mice (Figure 2B). As shown in Figures 2C, 6-m APPKI mice exhibited significantly reduced fear learning acquisition on day 1, with no significant difference measured on day 2 or 3. These results indicate early-stage neurodegenerative learning deficits in 6-m APPKI mice. At 12 m, APPKI mice exhibited a significant decline in freezing events throughout the whole test including day 1 learning, day 2 contextual memory, and day 3 cued memory recall (Figure 2D). While these data clearly suggest leaning deficit and contextual memory impairment, a decline in freezing events during cued memory may be because of the difference in the learning process, and a question needs to be addressed in future study.

**Figure 3:**
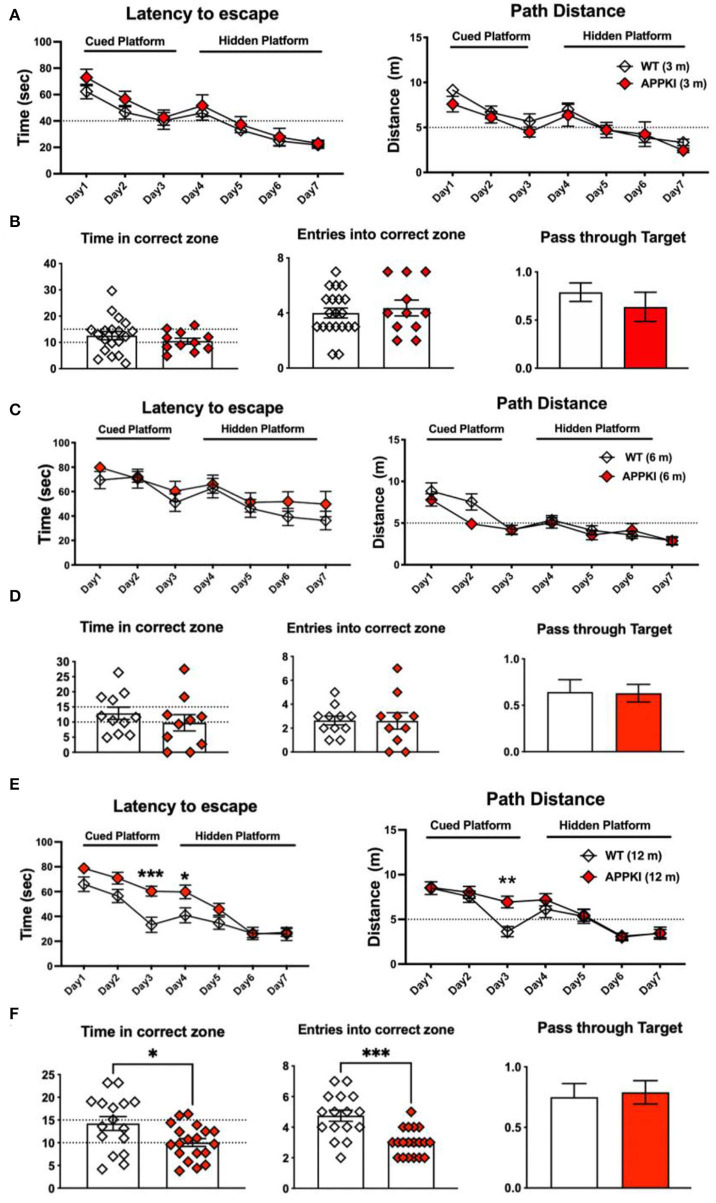
Learning and memory deficit of 3-, 6-, and 12-m APPKI mice by Morris water maze (MWM). **(A,C,E)** Results of days 1–7 showing time spent to reach platform and distance traveled to reach platform for 3-m, 6-m, and 12-m age of mice, respectively. Cued platform and hidden platform are both submerged underneath the water with or without a visible flag. **(B,D,F)** Results of day 8 showing the number of times mice entered the platform quadrant and the time spent in that zone, as well as the number of times passing through platform zone. Data are presented as mean ± SEM; *n* = 11–19 mice per group. Training session (days 1–7) was analyzed by two-way ANOVA followed by Bonferroni's multiple comparison test; probe session was analyzed by Student's *t*-test. Significance was assumed when *p* < 0.05. *p < 0.05, **p < 0.01, ***p < 0.005.

We also performed MWM to assess memory performance. As shown in Figures 3A,B, no significant change was observed in time and distance during MWM learning trials in 3-m APPKI mice. At 6 m, with the progression of the disease, APPKI mice took a slightly longer time and a longer distance to reach the platform when the platform was cued with a flag and hidden at days 1–7 compared with the age-matched WT (Figure 3C), and no significant change was observed in the probe test day (Figure 3D). At 12 months of age, APPKI mice significantly took a longer time and traveled a greater distance to reach the platform during days 1–7 compared with the age-matched WT (Figure 3E). At 12 m, APPKI mice also showed less time duration in the correct zone and fewer entries into the correct quadrant (Figure 3F).

### Neuroinflammation and Amyloidosis Phenotype in APPKI Mice

**Figure 4:**
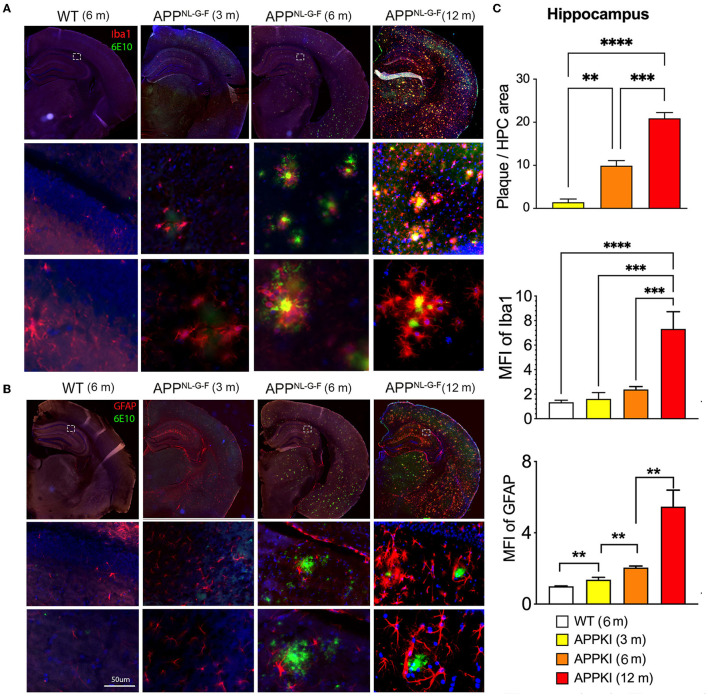
Age-dependent neuroinflammation and amyloidosis in APPKI mice. **(A)** Iba-1/6E10 and **(B)** GFAP/6E10 dual staining of APPKI mice brain at 3, 6–8, and 12–14 months. **(C)** Quantification of plaque load, Iba-stained microgliosis, and GFAP-stained astrocytosis in hippocampal region. *n* = 3–4 mice per group. A minimum of two sections from each mouse were used for quantitation. Data are presented as mean ± SEM and were analyzed using one-way ANOVA followed by LSD multiple comparison test. Significance was assumed when *p* < 0.05. **p < 0.01, ***p < 0.005, ****p < 0.001.

Since we have observed hippocampus-dependent learning and memory deficit, we thus performed immunofluorescence microscopy for neuroinflammation and amyloidosis and specifically quantized these markers in the hippocampal region. Aβ was stained with 6E10, microglia were identified by Iba-1, and astrocytes were stained with GFAP in brain sections. As expected, amyloid plaque deposition and neuroinflammation significantly progressed with age. While the accumulation of Aβ initially occurred at 3 m with astrocytes and encasing microglia (Figures 4A,B), prominent Aβ pathology was observed, especially in the piriform cortex, at 6 m. In contrast, less Aβ accumulation was observed in the hippocampus. In the hippocampus, Aβ accumulation is more enriched in CA3 and CA1, while the dentate gyrus is relatively spared. As shown in Figure 4C, activated microglia can be observed surrounding Aβ deposits in 3 m, while activated astrocytes surrounding Aβ deposit were significantly more pronounced in 6-m APPKI hippocampus. With the disease progress to the late stage, a robust accumulation of Aβ and over-reactive microglia and astrocytes was detected throughout the brain in 12-m APPKI mice.

### Hippocampal Mitochondrial Oxygen Consumption Rate (OCR) Significantly Decreases Over Time in APPKI Mice

**Figure 5:**
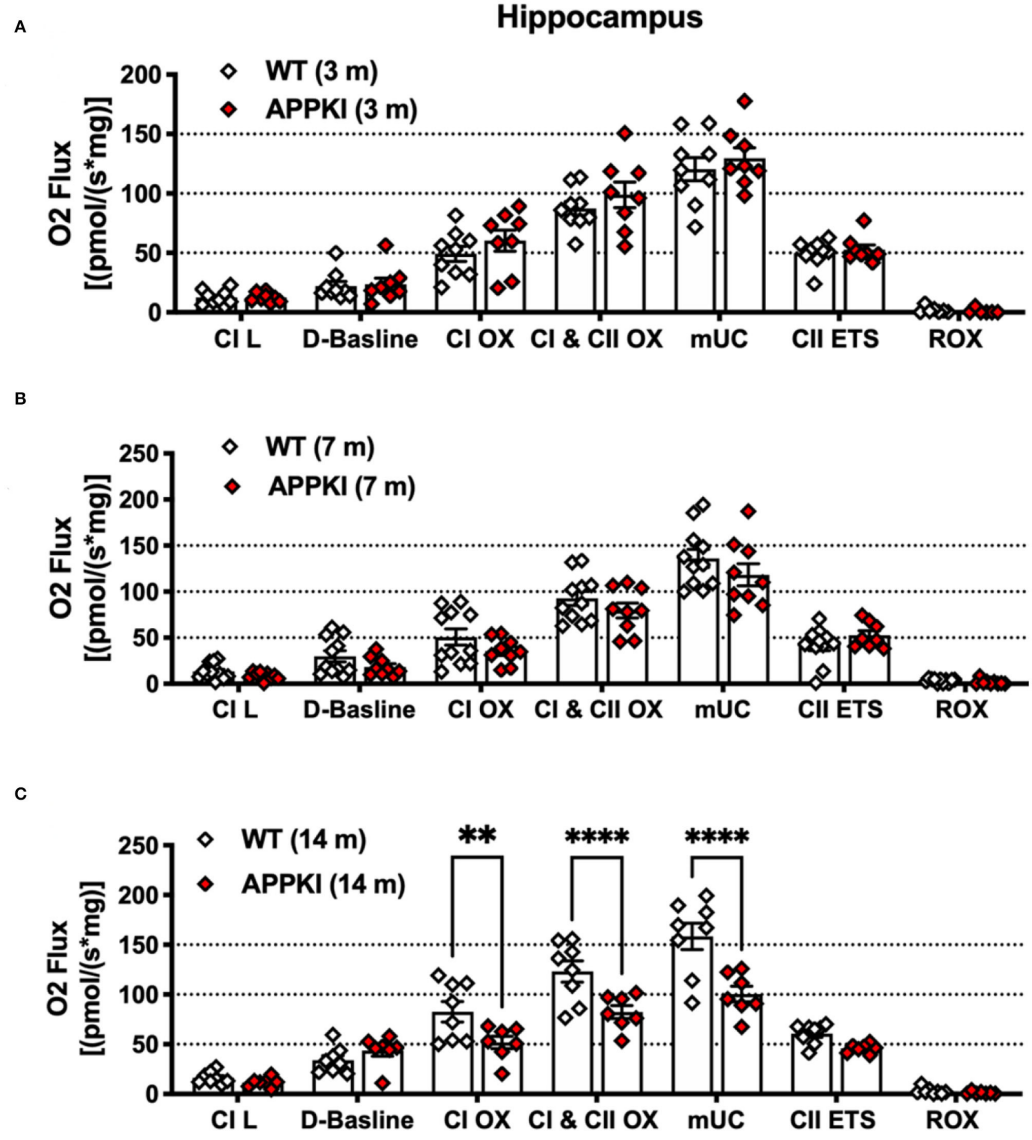
Mitochondrial respiration was decreased in 14-m APPKI hippocampal tissue. **(A–C)** Quantification of mass-specific oxygen flux from 3-m, 7-m, and 14-m APPKI and age-matched WT mice hippocampal homogenates. The horizontal bar denotes the respiratory states; L: Leak. Data are presented as mean ± SEM; *n* = 6–11 mice per group. Data were analyzed using one-way analysis of variance (ANOVA) followed by Bonferroni's multiple comparison test. Significance was assumed when *p* < 0.05. **p < 0.01, ****p < 0.001.

Mitochondrial alterations are closely associated with synaptic loss and neuronal death in AD. Mounting evidence has revealed that Aβ is a strong trigger for mitochondrial dysfunction in the CNS (Abramov et al., 2004; Mairuae et al., 2010; Leal et al., 2020). While the new APPKI mouse model has been well-characterized in terms of behavior and Aβ-related pathology, whether this model develops mitochondrial dysfunction and how this pattern affects disease progression has yet to be researched. We have thus examined hippocampal mitochondrial function in this study. Recent research has identified significant alternations in hippocampal mitochondrial morphology including increased mitochondria–endoplasmic reticulum (ER) contact sites (MERC) in the APPKI mice (Leal et al., 2020). Since we detected a low level of Aβ accumulation in the hippocampal region at 3 m, we therefore measured mitochondrial function using the high-resolution Oroboros O2k respirometer. As shown in Figure 5B, in the presence of glutamate, malate, and pyruvate, no significant difference was found in Leak state (L), complex I-driven (CI OX) respiration, complex II-driven respiration (CII ETS), ETS-independent residual oxygen consumption (ROX), maximal oxidative phosphorylation (CI and CII OX), and maximum uncoupled capacity (mUC) in response to FCCP titration in 3-m APPKI mice compared with age-matched WT mice (Figure 5A). At 7 m, a non-significant decrease trend was observed in complex I-driven (CI OX) respiration (*p* = 0.1041), maximal oxidative phosphorylation (CI and CII OX, *p* = 0.1569), and maximum uncoupled capacity (mUC, *p* = 0.0576) in response to FCCP titration in APPKI mice compared with age-matched WT mice (Figure 5B), while 14-m APPKI hippocampal lysate displayed significantly decreased mUC (*p* = 0.04) compared with age-matched WT mice (Figure 5C).

### Decreased ATP Production, Increased Mitochondrial Volume and Membrane Potential, and Reactive Oxygen Species (ROS) Production Rate in 7-m APPKI Hippocampal Mitochondria

**Figure 6:**
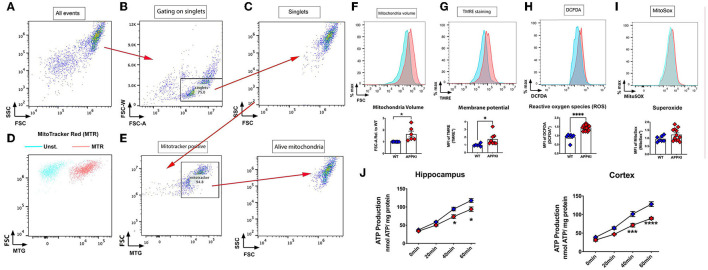
Mitochondrial dysfunction was observed in 7-m APPKI mice brain. **(A)** Isolated hippocampal mitochondria are visualized on a dot plot. **(B)** Doublets and singlets can be visualized by plotting FSC vs. FSC-W (linear mode). Doublets are excluded if the FSC-W value is more than two times the mean FSC-W value of the majority of events. Events under this threshold are gated as singlets. **(C)** Change the mode to dot plot again to visualize the gated singlet events to continue gating on the remaining events. **(D,E)** The unstained sample (blue events) and sample stained with a mitochondria-specific dye, MitoTracker Red (red events). Singlets were gated by MitoTracker and characterized for: **(F)** mitochondrial volume; **(G)** mitochondrial membrane potential; **(H)** ROS; **(I)** superoxide. **(J)** Decreased ATP production from purified mitochondria from hippocampal and cortical tissues. Data are presented as mean ± SEM; *n* = 3–4 mice per group with duplicates from each mouse. Data were analyzed using Student's *t*-test for F–I and two-way ANOVA for J. Significance was assumed when *p* < 0.05. *p < 0.05, ***p < 0.005, ****p < 0.001.

We next evaluated mitochondrial bioenergetics in concurrent experiments. The mitochondria isolated from one side of hippocampal tissue were incubated with MitoTracker, TMRE, Mito-SOX, and DCFDA to measure mitochondrial function as described in methods and Figures 6A–E. We did not observe any change in these mitochondrial function assays in 3-m APPKI mice. Similarly, no significant change was detected in ATP production from isolated hippocampal and cortical mitochondria in 3-m APPKI mice compared with WT mice (Supplementary Figure 1). However, at 7 months, APPKI mice hippocampal mitochondria exhibited a larger volume and a higher membrane potential (ΔΨm) compared with mitochondria from age-matched WT (Figures 6F,G), indicating increased polarization across mitochondrial membrane in APPKI mice. We also observed increased ROS production (Figure 6H), which is possibly caused by increased membrane potential (Abramov et al., 2010). Although no significant difference was found in superoxide production rate (Figure 6I), we did observe an increasing trend in APPKI mice compared with WT (*p* = 0. 1, two-tailed Student's *t*-test). We also measured ATP production using isolated mitochondria from hippocampal and cortical tissues in a separate set of experiments. As shown in Figure 6J, a significantly decreased ATP production was observed in mitochondria from both hippocampal and cortical tissues. The wide range of mitochondrial dysfunction observed in 7-m APPKI mice brain is consistent with previous findings showing an impaired metabolic profile precedes the clinical onset of AD (Kapogiannis and Mattson, 2011; Gordon et al., 2018).

### Significantly Decreased MFN2 Expression in 7-m APPKI Hippocampal Region

**Figure 7:**
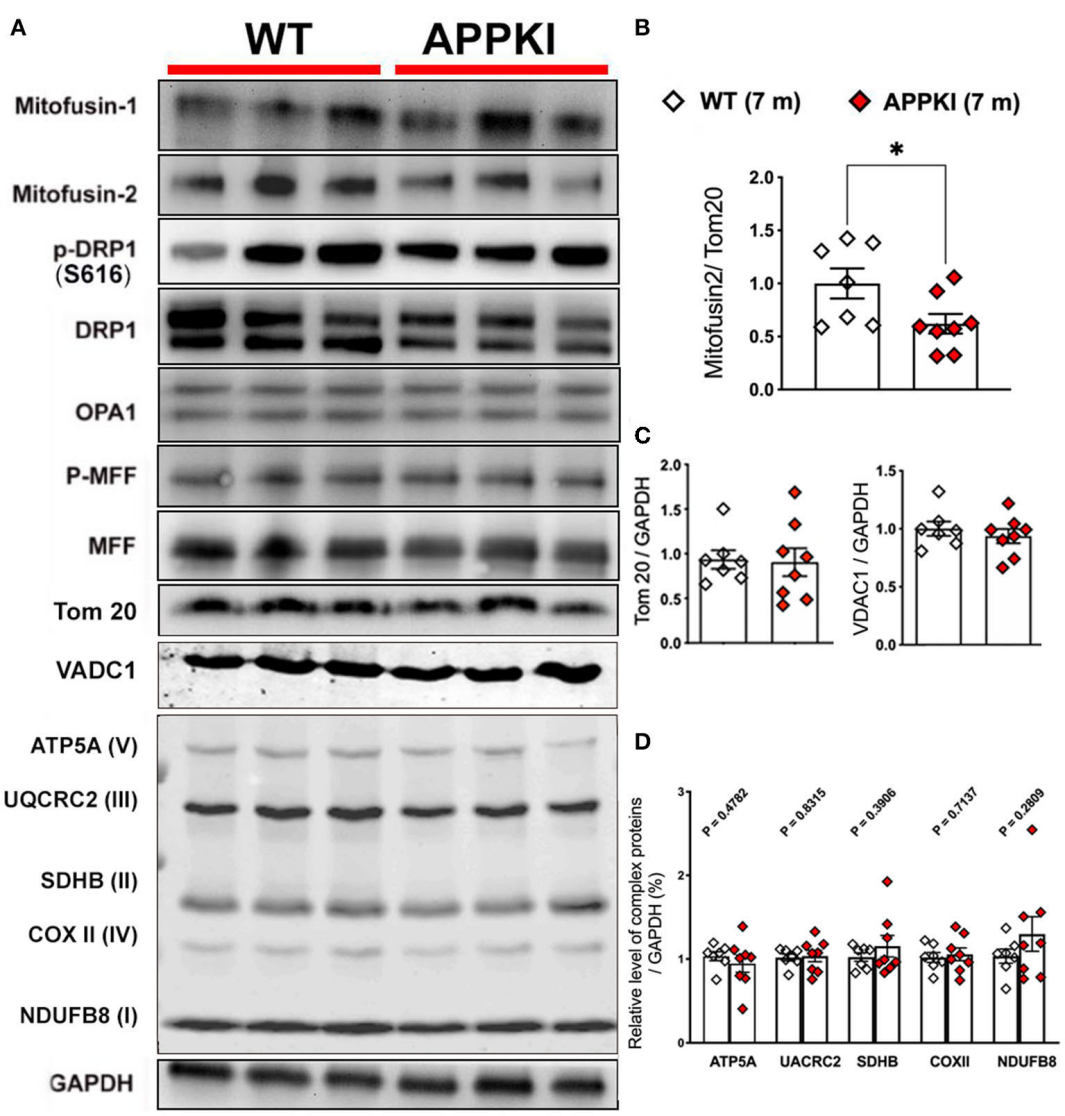
Decreased MFN2 in 7-m APPKI mice brain. **(A)** Representative blots of hippocampal homogenates from 7-m APPKI mice. **(B)** Densitometry analysis of MFN2 expression. Tom 20 was used as a loading control for all the mitochondrial dynamic proteins. **(C,D)** Densitometry analysis of Tom 20, VDAC1, and mitochondrial complex I–V expression. GAPDH was used as a loading control for **(C,D)**. Data are presented as mean ± SEM; *n* = 7–8 mice per group. Data were analyzed using Student's *t*-test, and significance was assumed when *p* < 0.05. *p < 0.05.

Mitochondria are dynamic organelles that undergo constant fission and fusion. The dynamic activity of mitochondria is maintained in a precise balance which is susceptible to pathophysiological changes such as Aβ accumulation and subsequent neuroinflammation. In the present study, we probed several mitochondrial dynamic-related proteins as shown in Figure 7A. Tom 20, the mitochondrial outer membrane protein, was used as a loading control for mitochondrial content. While most of mitochondrial dynamic proteins including MFN1, DRP, MFF, OPA1, and Tom 20 exhibit similar levels as WT, mitofusin-2 (MFN2, a mitochondrial fusion protein) significantly decreases in 7-m hippocampi homogenates compared with age-matched WT (Figures 7B,C). In addition, we also examined mitochondrial outer membrane protein VDAC1 and complex I–V (OXPHOS) expression in these 7-m APPKI mice hippocampal samples, and no significant difference was observed in APPKI mice compared with age-matched WT mice (Figure 7D). At the cellular level, a mitochondrial fusion activity safeguards mitochondrial quality control; the damaged mitochondria can be repaired by fusing with healthy mitochondria (Chen and Chan, 2009; Devireddy et al., 2015; Cai and Tammineni, 2016). It is possible that the decreased MFN2 at this early stage of AD contributed to the failure of mitochondrial quality control and contributed to the mitochondrial dysfunction observed at the same age, a hypothesis worthy of testing.

## Discussion

Of all the neuropathological changes observed in AD, synapse loss correlates most strongly with cognitive decline (Kashyap et al., 2019; Colom-Cadena et al., 2020). Mitochondria not only provide ATP for axonal transport and neurite growth, but synaptic-located mitochondria also provide energy that maintains synaptic function and mobilization of presynaptic vesicles essential for neurotransmission (Subramanian and Jonas, 2021). Thus, mitochondrial dysfunction in neurons can cause severe effects on synaptic activity and induce neuronal death and neurodegeneration. The present study used a newly developed preclinical AD mouse model (APPKI) to explore for the first time the mitochondrial bioenergetics and dysfunction caused by Aβ-related pathology, including (1) decreased ATP production, increased mitochondrial membrane potential, and ROS production and (2) downregulation of MFN2 protein level at the early stage of diseases parallel with cognitive deficit.

Although the general behavioral phenotype of APPKI mice has been described in many studies, most of the studies show alterations specifically in social and anxiety-related emotional domains (Sakakibara et al., 2018; Latif-Hernandez et al., 2019). Some observed mild learning and memory deficit in the APPKI mice at the late stage compared with the classical models that overexpress mutant APP (Sakakibara et al., 2018; Latif-Hernandez et al., 2019; Pervolaraki et al., 2019). The present study detected a significant deficit in the fear learning ability of APPKI mice as early as 6 m. Although no significant difference was detected in memory recall from FC at 6 m, we do observe impaired hippocampus-dependent spatial learning (day 1) at this stage. In contrast, Morris' water maze failed to detect any deficit at 6 m. The possible reason is that MWM involves many different paradigms such as motivation, motor activity, and vision besides cognition, and the learning deficits in 6-m APPKI mice are not sufficiently robust to be replicated in varying environments such as MWM. These data also point to the utmost importance of choosing the proper behavioral test for clinical trials, and the development of a consistent and sensitive diagnosis parameter in humans could significantly benefit early diagnosis and treatment. Taken together, the present study revealed a progressed cognitive degeneration of APPKI mice starts at 6 m, including learning deficit revealed by FC test and decreased exploration activity revealed by OF, an age that is slightly earlier than that presented by Masuda et al., which observed modest abnormalities in cognition in 8–9-m APPKI mice using machine learning (Masuda et al., 2016).

Mounting evidence shows that mitochondrial dysfunction plays a vital role in neurodegenerative diseases. Mitochondrial membrane potential (ΔΨm), which fuels ATP production, was found significantly decreased in neurodegenerative disease models (Schulz et al., 2012; Esteras et al., 2016). Membrane potential (ΔΨm) is created by the translocation of protons to the intermembrane space, which is coupled by the transfer of electrons from NADH and FADH_2_ to O_2_ through ETC. Surprisingly, the present study found that mitochondria isolated from 7-m APPKI hippocampi exhibit a significant increase in mitochondrial membrane potential. Interestingly, Delic et al. observed increased mitochondrial membrane potential in isolated brain mitochondrial from tauopathy mice and PSAPP mice (Delic et al., 2015; Hu et al., 2016; Dixit et al., 2017). Others have also shown increased mitochondrial membrane potential in iPSC-derived neurons of the C9ORF72 model of ALS/FTD (Lopez-Gonzalez et al., 2016) and iPSC-derived neurons from patients afflicted with frontotemporal dementia and parkinsonism linked to chromosome 17 (FTDP-17) (Esteras et al., 2017). Given that the mitochondrial membrane potential can affect overall energy production, it is likely that the oxygen being consumed may not be used toward ATP production but instead for ROS production. These data explain the unchanged OCR from 7-m APPKI hippocampi compared with age-matched WT.

Abnormalities in mitochondrial dynamics are widely observed in the setting of neurodegenerative conditions, including Alzheimer's disease and Parkinson's disease (PD) (Zhu et al., 2006; Jones, 2010; Schapira and Patel, 2014; Swerdlow, 2018; Tyumentsev et al., 2018), while under normal cellular physiological conditions, mitochondria undergo continuous fusion and fission to maintain a healthy pool of mitochondria with proper intracellular distribution. In the AD brain, both clinical and preclinical evidence has shown disrupted mitochondrial dynamics, including excessive fission and decreased fusion activity. This imbalanced dynamic change will result in failure of clearance of the damaged mitochondria and impaired axonal mitochondrial transport and exacerbate mitochondrial DNA (mtDNA) changes (Hirai et al., 2001; Manczak et al., 2018; Pickett et al., 2018; Wang et al., 2021a). The present study revealed a significant decrease in hippocampal MFN2 accompanied by a wide range of mitochondrial alterations from early-stage (6-m) APPKI mice. Importantly, other mitochondrial dynamic regulatory proteins such as MFN1, DRP, MFF, and OPA remained at levels similar to WT, together with unchanged mitochondrial complexes OXPHOS and outer membrane Tom20, VDAC1 expressions, indicating that the change in MFN2 expression may be the predominant contributing factor to the mitochondrial dysfunction observed at 6 m. However, it is noteworthy that evidence from both transgenic animal models and human postmortem brain tissue has reported these mitochondrial dynamic proteins are significantly altered in the later stage of Alzheimer's disease (Wang et al., 2009; Manczak et al., 2011; Batista et al., 2021). MFN2 is normally located on the outer mitochondrial membrane. As a main regulator for mitochondrial fusion activity, MFN2 plays an essential role in maintaining normal neuronal functions. Mounting evidence has shown downregulated MFN2 in the brain of rodent AD models (Wu et al., 2014; Tyumentsev et al., 2018) and postmortem AD patients (Manczak et al., 2011; Sita et al., 2020). In addition to its undisputed role in mitochondrial fusion regulation, MFN2 is also involved in mitochondria–ER association (Merkwirth and Langer, 2008), mitophagy (Sebastian et al., 2016), and axonal transport of mitochondria (Misko et al., 2010). A recent study has revealed that a lack of MFN2 can induce axonal degeneration and mitochondrial dysfunction in motor neurons *in vitro* (Mou et al., 2021). Additional *in vivo* evidence also demonstrated that loss of MFN2 during adulthood causes an oxidative stress response and neuronal death, ultimately resulting in learning and memory deficits (Jiang et al., 2018; Ishikawa et al., 2019). Thus, it is possible that the significant decrease in hippocampal MFN2 measured in 7-m APPKI mice may provide a molecular mechanism for the failed bioenergetics in these mice. In the last few years, MFN2 in the pathogenesis of AD has become a research hot spot (Kim et al., 2017; Leal et al., 2020; Sita et al., 2020; Wang et al., 2021), and the present study provides a strong support for the hypothesis that MFN2 may serve as a therapeutic target for AD.

In summary, our study not only details behavioral impairments together with deposition of amyloid plaque and neuroinflammation and disrupted mitochondrial bioenergetics in an age-dependent manner, which strongly support the suitability of APPKI mice in the research of AD, but our result also suggests mitochondrial dysfunction as a very early event in the AD pathogenesis and proposed a potential therapeutic target, MFN2, for the treatment of AD. The detailed behavioral and bioenergetic alterations described in this physiologically relevant AD transgenic mouse model provide a valuable tool to optimize the temporal component for therapeutic interventions to treat AD.

## Data Availability Statement

The original contributions presented in the study are included in the article/Supplementary Material, further inquiries can be directed to the corresponding author/s.

## Ethics Statement

All animal protocols were reviewed and approved by the Veterans Administration San Diego Healthcare System Institutional Animal Care and Use Committee (#20-030).

## Author Contributions

SW performed all experiments, analyzed the data, and wrote and edited the manuscript. TI performed the mitochondrial function assessment. SD performed the Oroboros assay and analyzed the data. PS performed the colony maintenance and ATP assay. GB performed the behavioral tests. HP assisted with the Oroboros data interpretation. BH guided the overall study design, data interpretation, and scientific concept and wrote and edited the manuscript. All authors read and approved the final manuscript.

## Funding

The work in the authors' laboratories was supported by Veterans Affairs Merit Award from the Department of Veterans Affairs BX003671 (BH) and BX005229 (HP).

## Conflict of Interest

The authors declare that the research was conducted in the absence of any commercial or financial relationships that could be construed as a potential conflict of interest.

## Publisher's Note

All claims expressed in this article are solely those of the authors and do not necessarily represent those of their affiliated organizations, or those of the publisher, the editors and the reviewers. Any product that may be evaluated in this article, or claim that may be made by its manufacturer, is not guaranteed or endorsed by the publisher.
