# Supplementary material for: Age-Dependent Behavioral and Metabolic Assessment of AppNL−G−F/NL−G−F Knock-in (KI) Mice
Source: Front Mol Neurosci. 2022 Jul 29;15:909989. doi: 10.3389/fnmol.2022.909989 (PMC9373872; doi:10.3389/fnmol.2022.909989)
Supplement: Supplementary file 1 [file Image_1.pdf]

Supplementary Material

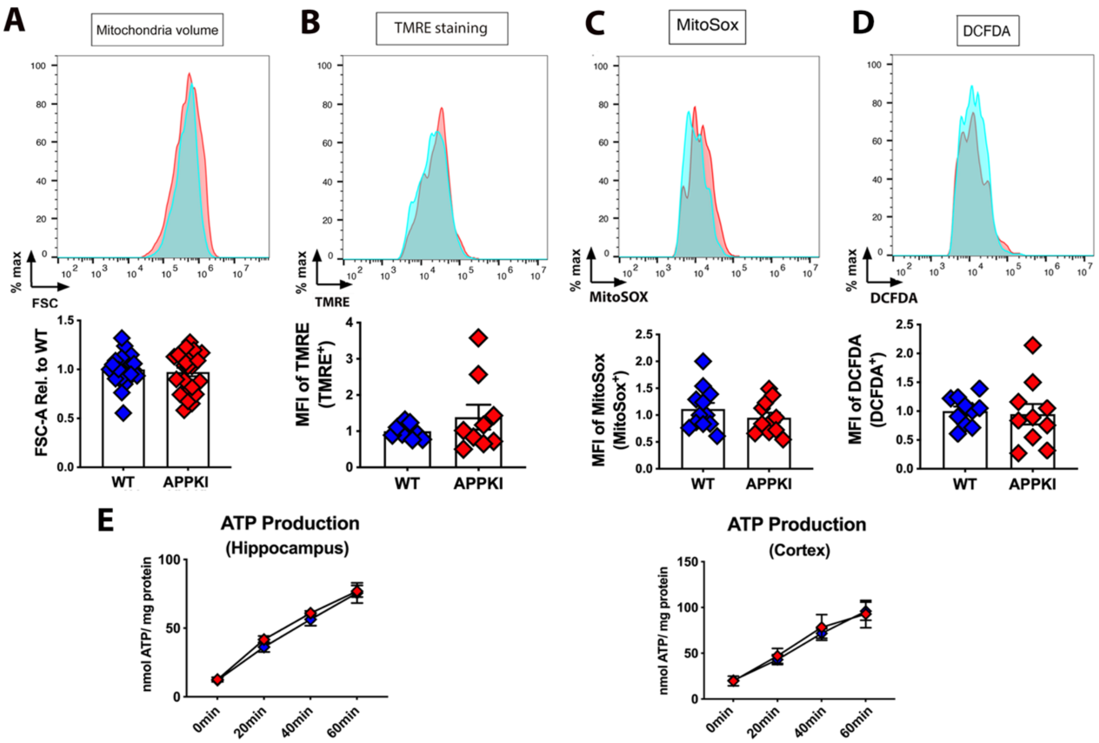

Supplementary Figure 1. No deficit in mitochondria function was observed in 3 m APPKI mice brain. Characterization of purified mitochondria from 3 m WT and APPKI mice hippocampal tissue. **(A)** Mitochondrial volume; **(B)** Mitochondrial membrane potential; **(C)** Superoxide; **(D)** ROS. **(E)** ATP production of mitochondria isolated from hippocampal and cortical tissue. Data are presented as mean ± S.E.M, n = 4-5 animals per group with minimal of duplicates samples from each animal. Data were analyzed using t-test or Two-Way ANOVA. Significance was assumed when $p < 0.05$.
